# Supplementary material for: The interactome of intact mitochondria by cross-linking mass spectrometry provides evidence for coexisting respiratory supercomplexes
Source: Mol Cell Proteomics. 2017 Dec 8;17(2):216–32. doi: 10.1074/mcp.RA117.000470 (PMC5795388; doi:10.1074/mcp.RA117.000470)
Supplement: Supplemental Data [file supp_RA117.000470_133922_0_supp_23978_fzffwf.zip › spectra_annotation/mito_DR_spectra_annotation/91-1-2-1-13-1.pdf]

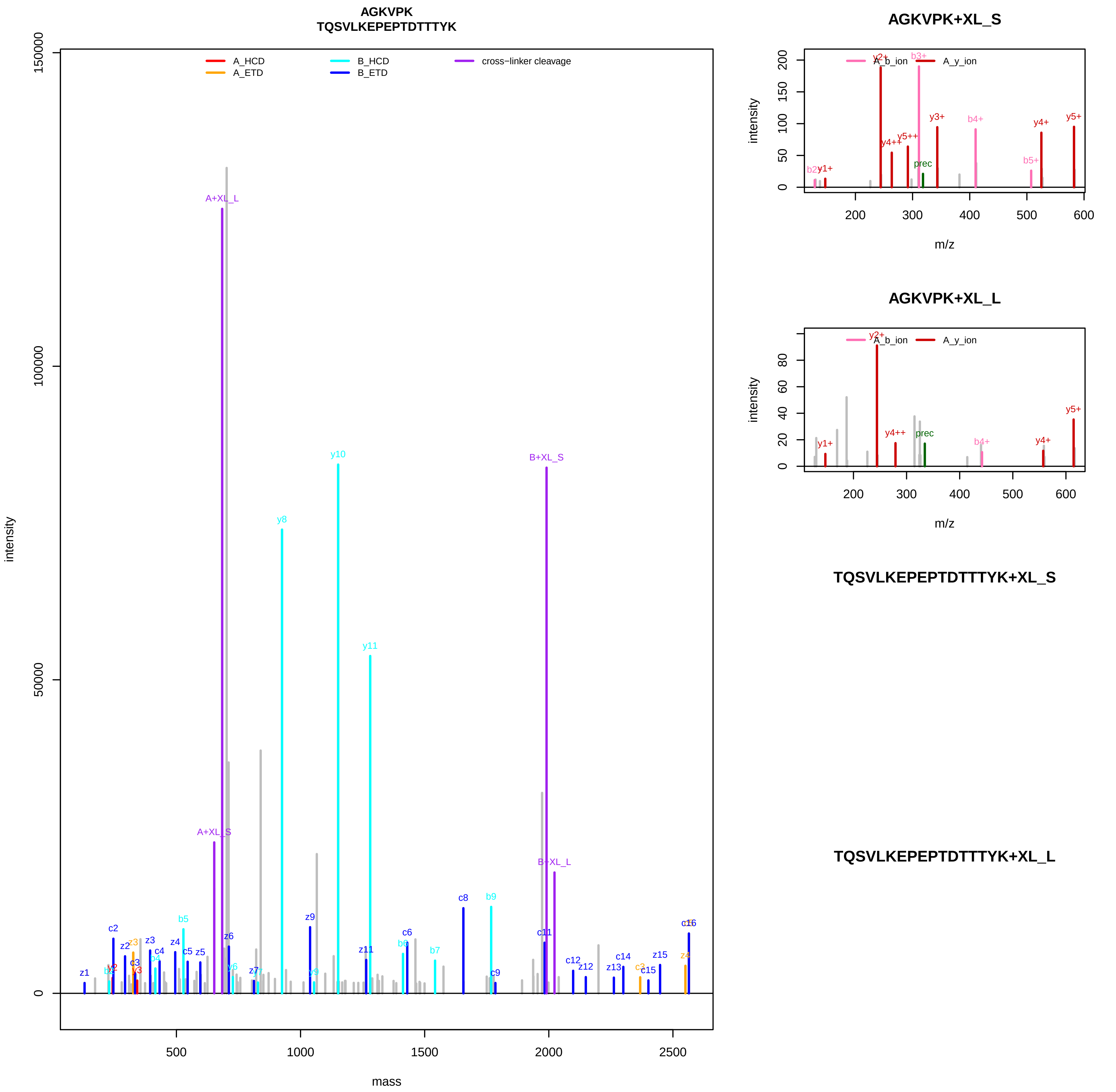
AGKVPK
TQSVLKEPEPTDTTTYK
A_HCD
A_ETD
B_HCD
B_ETD
cross−linker cleavage
intensity
mass
A+XL_L
A+XL_S
B+XL_S
B+XL_L
y8
y10
y11
b5
b7
b9
c2
c4
c5
c6
c8
c9
c11
c12
c14
c15
c16
z1
z2
z3
z4
z5
z6
z7
z9
z11
z12
z13
z15
AGKVPK+XL_S
AGKVPK+XL_L
A_b_ion
A_y_ion
prec
y1+
y2+
y3+
y4+
y5+
y4++
y5++
b2+
b3+
b4+
b5+
m/z
TQSVLKEPEPTDTTTYK+XL_S
TQSVLKEPEPTDTTTYK+XL_L
